# At-Home Transcranial Direct Current Stimulation (tDCS) With Telehealth Support for Symptom Control in Chronically-Ill Patients With Multiple Symptoms

**DOI:** 10.3389/fnbeh.2018.00093

**Published:** 2018-05-22

**Authors:** Alexa Riggs, Vaishali Patel, Bhaskar Paneri, Russell K. Portenoy, Marom Bikson, Helena Knotkova

**Affiliations:** ^1^MJHS Institute for Innovation in Palliative Care, New York, NY, United States; ^2^Department of Biomedical Engineering, Grove School of Engineering, The City College of New York, New York, NY, United States; ^3^MJHS Hospice and Palliative Care, New York, NY, United States; ^4^Department of Family and Social Medicine, Albert Einstein College of Medicine, New York, NY, United States; ^5^The Saul R. Korey Department of Neurology, Albert Einstein College of Medicine, New York, NY, United States

**Keywords:** non-invasive brain stimulation, transcranial direct current stimulation, patient-tailored protocol, chronic illness, symptom management, home settings, at-home tDCS

## Abstract

Transcranial direct current stimulation (tDCS) delivered in multiple sessions can reduce symptom burden, but access of chronically ill patients to tDCS studies is constrained by the burden of office-based tDCS administration. Expanded access to this therapy can be accomplished through the development of interventions that allow at-home tDCS applications.

**Objective:** We describe the development and initial feasibility assessment of a novel intervention for the chronically ill that combines at-home tDCS with telehealth support.

**Methods:** In the developmental phase, the tDCS procedure was adjusted for easy application by patients or their informal caregivers at home, and a tDCS protocol with specific elements for enhanced safety and remote adherence monitoring was created. Lay language instructional materials were written and revised based on expert feedback. The materials were loaded onto a tablet allowing for secure video-conferencing. The telehealth tablet was paired with an at-home tDCS device that allowed for remote dose control via electronic codes dispensed to patients prior to each session. tDCS was delivered in two phases: once daily on 10 consecutive days, followed by an as needed regimen for 20 days. Initial feasibility of this tDCS-telehealth system was evaluated in four patients with advanced chronic illness and multiple symptoms. Change in symptom burden and patient satisfaction were assessed with the Condensed Memorial Symptom Assessment Scale (CMSAS) and a tDCS user survey.

**Results:** The telehealth-tDCS protocol includes one home visit and has seven patient-tailored elements and six elements enhancing safety monitoring. Replicable electrode placement at home without 10–20 EEG measurement is achieved via a headband that holds electrodes in a pre-determined position. There were no difficulties with patients’ training, protocol adherence, or tolerability. A total of 60 tDCS sessions were applied. No session required discontinuation, and there were no adverse events. Data collection was feasible and there were no missing data. Satisfaction with the tDCS-telehealth procedure was high and the patients were comfortable using the system.

**Conclusion:** At-home tDCS with telehealth support appears to be a feasible approach for the management of symptom burden in patients with chronic illness. Further studies to evaluate and optimize the protocol effectiveness for symptom-control outcomes are warranted.

## Introduction

In the United States, at least 175 million people are chronically ill (Centers for Medicare and Medicaid Services, 2012; Ortman et al., 2014). Most are elderly and receive care in the ambulatory setting or at home (Hasselman and Center for Health Care Strategies, 2013). Effective symptom management is essential but is often challenged by the occurrence of multiple symptoms and the need for polypharmacy, which may produce adverse effects in the medically fragile. For many patients, the need for frequent visits to a physician’s office presents additional challenges. Innovative strategies for symptom management that could be provided at home and reduce the reliance on drug therapy would represent a significant advance in addressing the burden of illness among seriously ill patients.

Transcranial direct current stimulation (tDCS) is a non-invasive and non-pharmacological intervention that may address multiple symptoms of chronic illness (Nitsche and Paulus, 2000; Fregni et al., 2006a,b; Rigonatti et al., 2008; Valle et al., 2009; Loo et al., 2012; Knotkova et al., 2014a,b). tDCS is delivered via a battery-powered device with two or more electrodes that transfer electrical current of low intensity (usually 1–2 mA) to the surface of the head. The primary mechanism of tDCS is a subthreshold modulation of neuronal resting membrane potential (Nitsche and Paulus, 2000). If delivered for several minutes, tDCS can induce neuroplasticity of glutamatergic synapses, resulting in enduring alterations of neural excitability (Nitsche et al., 2003; Antal et al., 2010; Monte-Silva et al., 2013). Recent evidence suggests that tDCS interacts with various neurotransmitters in the brain, such as dopamine, acetylcholine, serotonin and gamma-aminobutyric acid (GABA; Nitsche et al., 2004a,b,c, 2006, 2009; Polania et al., 2011; Stefani et al., 2012; Stagg et al., 2013). The neurophysiologic effects of tDCS can be detected in cortical and subcortical areas distant from the site of stimulation, and recent findings suggest that tDCS can upregulate and downregulate functional connectivity within complex brain networks, such as those that are important for cognitive, motor, and pain processing or mood modulation (Stagg et al., 2013). The scientific rationale for tDCS studies in symptom management builds on findings from neurophysiological and neuroimaging studies indicating that the development and maintenance of symptoms frequently occurring in various chronic illnesses are associated with changes in cerebral networks and altered functional connectivity (e.g., Apkarian et al., 2013; Hemington et al., 2016). Earlier studies suggest that reversal of these potentially maladaptive changes in brain function is possible and can be associated with an improvement of symptoms (Maihöfner et al., 2003, 2004; Napadow et al., 2012; and others). Neuromodulation techniques, such as tDCS, can induce enduring alterations of neural activity and connectivity, and can be used to attempt reversal the maladaptive neuroplastic changes occurring in chronic illness (Lefaucheur, 2016; Stock et al., 2016; Woods et al., 2016).

Numerous randomized controlled trials suggest that tDCS can relieve symptoms common in chronic illness, such as pain, fatigue, sleep difficulties, or mood disturbance (Fregni et al., 2006a,b; Roizenblatt et al., 2007; Boggio et al., 2008; Valle et al., 2009; Loo et al., 2012; Brunoni et al., 2014; Ferrucci et al., 2014; Saiote et al., 2014; Fagerlund et al., 2015; and others). Although the extant evidence would support a trial of tDCS in chronically ill patients with multiple poorly controlled symptoms, the access to tDCS for those patients has been constrained. There are many potential reasons for this observation, among which may be the burden associated with the conventional tDCS approach, which requires consecutive daily visits to an office for 1 or more weeks. The development of a home-based tDCS intervention suitable for the chronically ill with complex symptoms would have the potential to expand access to this promising non-pharmacological method.

We have developed a novel intervention that combines at-home tDCS and telehealth support; we have adapted the tDCS procedure and technology for easy home use by the chronically ill, and developed instructional materials, a comprehensive training plan, and a patient-tailored tDCS application protocol that allows for dose-control, and enhanced adherence and safety monitoring in remote. Here we describe the development of this intervention and feasibility testing in four patients with advanced chronic illness and multiple unrelieved symptoms.

## Materials and Methods

### Development

The guiding principles for the development of the intervention were low burden for the patient, strict dose control, and enhanced monitoring for adherence and safety. To identify and adjust potentially difficult steps in the tDCS application for patients and their informal caregivers, analysis of the tDCS procedure was performed. Possible solutions were presented to a group of hospice and palliative care professionals in expert-feedback sessions that included in total six experts (1M, 5F) whose experience in hospice and palliative care averaged 24 years and whose current positions were either managerial or clinical. After each expert provided consent, he or she was interviewed and asked to provide specific feedback on suitability of the adjusted tDCS procedure for patients and their informal caregivers and on versions of lay-language training materials – an instructional video and a step-by-step brochure.

The device for home-based tDCS was the “1X1 tDCS mini-CT” device, model 1601 (Soterix Medical Inc., NY, United States), with two 5 cm × 5 cm saline-soaked sponge electrodes. The device included a safeguard for dose control, consisting of electronic unlocking codes, that were provided to the patient or caregiver prior to each tDCS treatment. The device also generated an electronic post-treatment record that informed the tDCS technician about completion, interruption, or restart of the tDCS session.

The telehealth device paired with the tDCS unit was a Samsung Galaxy touchscreen tablet 6.93^′′^ × 9.57^′′^. The tablet contained educational and instructional materials for the patient and informal caregiver and allowed secure videoconferencing with the patient for remote assistance and adherence monitoring by the tDCS technician.

### Pilot to Assess Feasibility

To perform an initial evaluation of the feasibility of the at-home tDCS-telehealth intervention, four patients were recruited following referrals from MJHS Hospice and Palliative Care personnel. Patients were required to be at least 18 years of age, English-speaking, able to provide informed consent, have at least one chronic illness, and to report moderate to severe distress from one or more of the following symptoms: pain, fatigue, difficulties concentrating, worrying, feeling sad, or feeling nervous, as self-rated by the patient on the Condensed Memorial Symptom Assessment Scale (CMSAS; below).

Patients were excluded if they had metal implants in the head or in the neck; cancer affecting the head; history of seizures or seizure disorder; unstable acute medical condition; compromised integrity or sensitivity of the skin at or near locations where electrodes would be placed; or if they used another neurostimulation device.

Patients had an option to include their informal caregiver to assist with the tDCS procedures. To co-participate, the caregivers were required to be at least 18 years of age, able to follow instructions in English, and to provide informed consent.

The at-home tDCS was applied in accordance with a patient-tailored protocol (described in detail in section Results) in two phases: 10 daily tDCS sessions on 10 consecutive days were followed by *as needed* applications over 20 days, using either the “DLPFC” montage or “M1-SO” electrode montage. The montage selection was congruently informed by findings from previous studies and patient’s report of most distressing symptom(s) at screening. Patients were considered for the study participation if they reported moderate to severe distress from one or more of the following symptoms: worrying, feeling sad, feeling nervous, difficulties concentrating, fatigue, or pain. Based on findings from previous studies, each of these symptoms can be addressed using the “DLPFC” montage with the anode on the left hemisphere over the area corresponding with the F3 point of the international 10–20 EEG system and the cathode over F4 (Valle et al., 2009; Brunoni et al., 2013; Saiote et al., 2014; Sandrini et al., 2014; Glaser et al., 2016; Manenti et al., 2017; and others). However, specifically for pain, the more common tDCS montage is the “M1-SO” montage, with the anode over the area of the motor cortex (C3 or C4 of the 10–20 EEG system) contralateral to pain-affected side of the body in unilateral pain, or over C3 in bilateral pain, with the cathode over the supraorbital region on the hemisphere contralateral to the anode placement (Fregni et al., 2006a,b; Valle et al., 2009; Mori et al., 2010; Kim et al., 2013; and others). In our study, the “M1-SO” montage was used if pain was reported as dominating most distressing symptom.

#### Data Collection

Patients’ sociodemographic characteristics – age, race, marital status, living status (lives alone, does not live alone), education (high school ∼12 years of education; some college ∼13–15 years; undergraduate ∼16 years; graduate school ∼18+ years of education), were recorded. Performance status was assessed with the Karnofsky Performance Status scale (KPS) between 0 and 100, with anchor points 0 (death) and 100 (no complaints/no evidence of disease) (Schag et al., 1984). Symptom distress was assessed with the CMSAS, a validated 14-item questionnaire used to evaluate the prevalence and distress associated with 11 physical and 3 psychological symptoms (Chang et al., 2004). Prevalence was indicated on a single item, which was checked if the symptom was experienced during the indicated timeframe. Distress due to each of the physical symptoms was measured on a Likert-type scale, which asked “How much did the symptom bother or distress you in the past 7 days?” and was graded using a 5-point scale: 0 (no distress at all), 1 (a little bit), 2 (somewhat), 3 (quite a bit), and 4 (very much). Distress due to each of the psychological symptoms was measured on a 4-point scale that asked “How frequently did the following symptom occur?” and was graded 1 (rarely), 2 (occasionally), 3 (frequently), 4 (almost constantly). The scores for the Global Distress and its two subscales Physical Symptom Distress and Psychological Symptom Distress were calculated as the sum of the scores reported on corresponding groups of physical and psychological symptoms. The assessment was carried out at the baseline, at the end of the first ten sessions, and again at the end of the twenty-day optional phase involving up to ten additional applications. Patient satisfaction with the tDCS system was determined from an 8-item user survey administered after the last tDCS application.

The study was carried out in accordance with the recommendations of the Handbook for Good Clinical Research Practice, WHO, 2002. The procedures were approved by the New England Independent Review Board (NEIRB), and all participants provided written informed consent in accordance with the Declaration of Helsinki.

## Results

### Development of the Intervention

#### Adjustment of the tDCS Application Process

A step-by-step analysis of tDCS application process informed by expert feedback identified major challenges associated with preparation of the electrodes and with the electrode montage (**Table 1**).

**Table 1:** Analysis of the tDCS application process, with focus on potentially difficult steps in tDCS application for seriously ill patients and their informal caregivers; and possible solutions.

Step	Challenge	Solution
Having all elements of the tDCS kit ready	Misplacing supplies	Supplies for one stimulation session enclosed in an individual pouch; sufficient number of pouches included in the tDCS-kit briefcase dispensed to patient at the initial visit; the tDCS-kit briefcase labeled, with a note for proper storage.
Preparing electrodes	Proper assembly of the electrode	Cable insertion clearly depicted in the patient’s instructional materials
	Replicable saturation	5 ml syringes pre-filled with saline included in the tDCS kit
Electrode montage	Determining the electrode position without the 10–20 EEG measurements	Size-fitted headband allowing for accurate and replicable electrode placement (**Figure 1**)
	Securing the electrode on the head	Size-fitted headband
Connecting electrode cables with the device	No challenge (cables and inserts are color-coded)	–
Preparing the device	Identification of the keypad buttons	Depicting the keypad and snapshot of the screen in the patient’s instructional materials
Checking connection	Understanding connection-quality grades	Using lay-language words; avoiding alarming words, such as “critical”
	Performing corrective action if connection quality is unacceptable	Training the patient in possible corrective actions
Starting the stimulation	Using the start code obtained from tDCS technician	Describing the procedure in lay language in the patient’s instructional materials and depicting the snapshot of the screen and the prompt for the start code
Ending the stimulation	Obtaining/recording the end code generated by the device	Describing the procedure in lay language in the patient’s instructional materials and depicting an example of the generated end-code
Clean up	No challenge	–
Batteries	Changing/charging batteries as needed	Depicting the process step-by-step in the patient’s instructional materials

**FIGURE 1:**
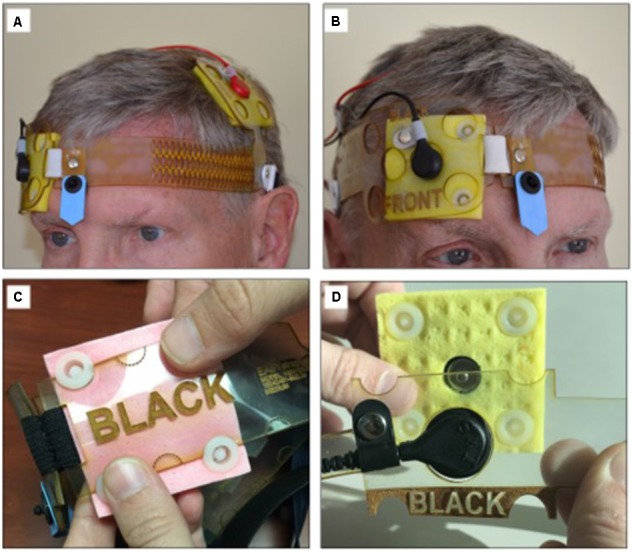
tDCS headgear for the electrode M1-SO montage with the anode over the area of the motor cortex and the cathode over the contralateral supraorbital region. The headgear **(A**,**B)** does not require neuronavigational measurements. The center point (blue arrow) supports accurate self-placement by user. The headgear accommodates either conventional saline-soaked electrodes **(C)** or snap-on pre-moisturized ones **(D)**. Panel AB is courtesy of HK. The person shown has given permission to publish this picture.

To assure replicable saturation of the electrode sponges, saline was pre-filed into 5 ml syringes that were included in the tDCS kit dispensed to the patients. To achieve electrode montage without the 10–20 EEG measurements, electrodes were placed in a headband that held the electrodes in the desired position for the stimulation over the area of the primary motor cortex (“M1-SO” montage, **Figure 1**; Knotkova et al., 2017), or over the dorsolateral prefrontal cortex (“DLPFC”; Seibt et al., 2015), based on reported symptoms. The headband had a navigational mark that served as reference point for proper positioning and was size-fitted to the patient at the time of equipment dispense.

#### Patient-Tailored Protocol

**FIGURE 2:**
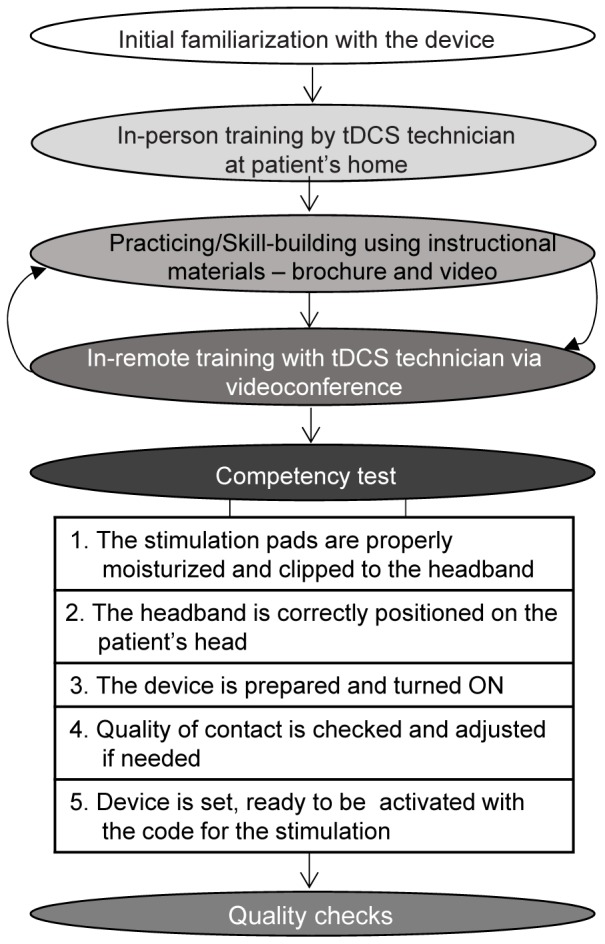
A step-by-step training plan to build patients’ and caregivers’ competency for tDCS applications.

**FIGURE 3:**
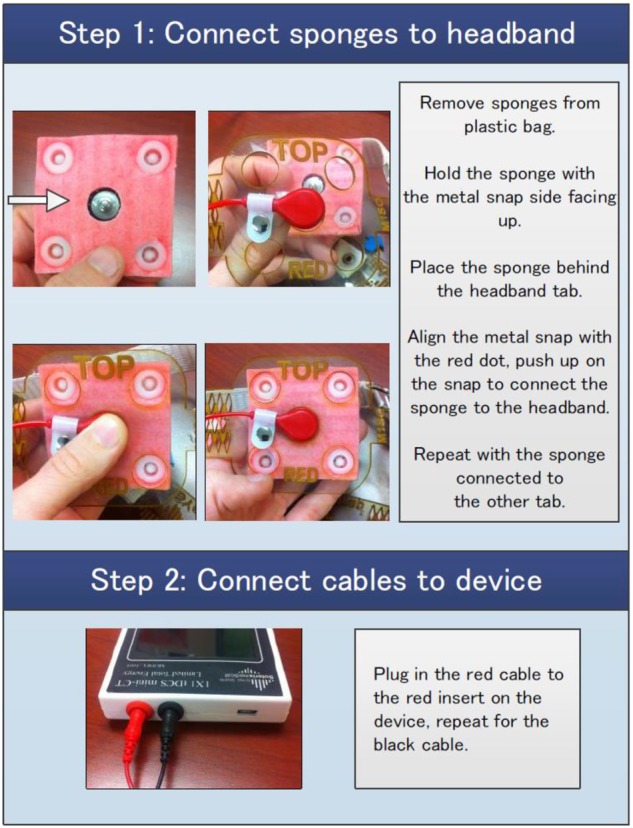
A snapshot of the tDCS instructional brochure for patients.

The protocol included one home visit for consenting, eligibility screening, initial tDCS tolerability test, and in-person initiation of tDCS training. The training process (**Figure 2**) continued using instructional materials preloaded on the telehealth tablet (**Figure 3**) with remote assistance via videoconference as needed. The training was concluded with the tDCS Competency test. tDCS was applied in two phases; first, patients are encouraged to apply 10 sessions, one 20-minute stimulation per day on 10 consecutive days. This build-up phase was followed by a fully optional 20-day period, during which the patient could apply tDCS as needed for up to ten additional sessions delivered once or twice per day. The number of applications in the “as-needed” regimen was guided by the patient judgment and the frequency was determined by the patient without input from the study personnel.

**FIGURE 4:**
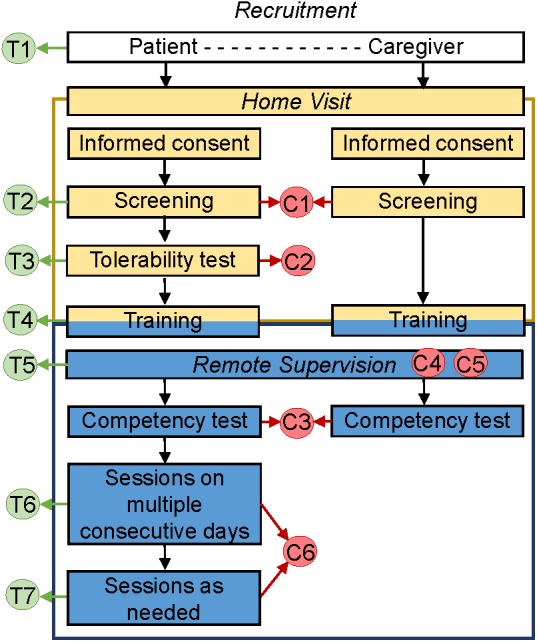
Protocol for tDCS application by chronically ill patients at home. The protocol includes 1 at-home visit and has specific elements that allow for tailoring to the needs and preferences of the patient (T1–T7), as well as elements that support safety and adherence monitoring (C1–C6).

The protocol had seven specific elements that allowed for tailoring (T) to the needs and preferences of the patient (**Figure 4**, T1–T7), and six elements that supported monitoring for safety and compliance with the protocol (C; **Figure 4**, C1–C6).

The elements for tailoring treatment included: (1) optional inclusion of assisting informal caregiver; (2) broad inclusion criteria that allow targeting of one or more of multiple symptoms; (3) tailored stimulation intensity (default of 1.5 mA, but able to be decreased to 1 mA for patients who perceive 1.5 mA as unpleasant); (4) tailored tDCS training involving either the patient or a co-participating informal caregiver, or both, and mediated through instructional video, videoconference with tDCS technician and/or a step-by-step brochure; (5) versatility of remote assistance via a videoconference or by phone, with frequency and mode of assistance determined by the patient’s needs; (6) a recommended regimen during the first block of stimulation (tDCS applications on consecutive days strongly encouraged but not mandated); and (7) optional applications in the second phase, potentially allowing twice daily stimulation.

The elements that supported safety and monitoring for the adherence with the protocol (**Figure 4**, C1–C6) included the following: (1) exclusion criteria that precluded tDCS initiation in patients with metal implants in the head/face, history of seizures or brain tumor, compromised skin integrity at the electrode-placement area, or an inability to provide consent or follow instructions; (2) initial tDCS tolerability test, which excluded participants who could not tolerate 1 mA of stimulation; (3) use of a competency test at the conclusion of training to assure preparedness of the tDCS operator (patient or assisting informal caregiver), and exclusion of patient if competency in the application process could not be demonstrated within 1 week after initiation of training; (4) monitoring for compliance with good practices in tDCS (including remote quality checks to evaluate if procedures for the electrode preparation and tDCS delivery were done properly and in accordance with training materials); (5) comprehensive safety monitoring that ensured that safety checks prior to and during stimulation were completed (e.g., intact skin in the electrode area and absence of adverse effects), and that stimulation be stopped if perceived as painful; and (6) electronic dose control: the device was electronically unlocked for each session at a pre-set dose and provided an end-of-session code indicating whether stimulation was completed, delivered without or with interruptions, or was aborted.

### Pilot to Assess Feasibility

Four patients were recruited for the initial feasibility assessment. The patients’ characteristics are summarized in **Table 2**.

**Table 2:** Patients’ profiling characteristics.

Profiling characteristics	Patients			
	1	2	3	4
Age	52	44	58	63
Race/ethnicity	Hispanic	Hispanic	White	Hispanic
Sex	Male	Female	Female	Male
Marital status	Single	Single	Married	Married
Living status	Doesn’t live alone	Doesn’t live alone	Doesn’t live alone	Doesn’t live alone
Employment status	Not employed	Not employed	Not employed	Not employed
Education in years	13–15	13–15	18+	12
Karnofsky performance status	30	80	90	70

Patient 1 was a 52-year-old Hispanic male, single, but not living alone, and not employed. He had advanced congenital myasthenia gravis associated with quadriplegia and chronic ventilator support. The patient experienced severe distress from chronic pain of the neck, lower back, and right lower limb for longer than 1 year. Other reported symptoms included fatigue, insomnia, difficulty concentrating, nervousness, worrying, feeling sad, lack of appetite, dry mouth, weight loss, and constipation. Symptoms were poorly controlled despite treatment with fentanyl and gabapentin. The patient was severely disabled, with the KPS score of 30.

Patient 2 was a 44-year-old Hispanic female, single, but not living alone, and not employed. She had a longstanding indolent spinal tumor associated with back pain and depressed mood (worrying, feeling sad), and reported severe distress from each of these symptoms for longer than one year. Other reported symptoms included lack of energy, lack of appetite, dry mouth, feeling drowsy, constipation, nausea, difficulties sleeping, and difficulties concentrating. Symptom-directed medications included baclofen, hydromorphone, oxycodone-acetaminophen, escitalopram, docusate, diphenhydramine, and pantoprazole sodium. The patient had the KPS score of 80, able to carry on normal activity with effort.

Patient 3 was a 58-year-old white female, married, and not employed. She had post-traumatic plexopathy and chronic pain in the left lower limb that caused moderate symptom distress for more than 1 year. Other reported symptoms were difficulty concentrating and feeling nervous. Pain was poorly controlled despite hydromorphone. Her KPS was 90, able to carry on normal activity.

Patient 4 was a 63-year-old Hispanic male, married, and not employed. The patient had a history of stroke and associated left leg and arm paralysis and chronic pain. The pain was adequately controlled with gabapentin, but the patient experienced burden due to psychological symptoms – worrying, feeling sad, and feeling nervous for longer than three months. His KPS was 70, able to care for himself, but not able to carry on normal activity or do active work.

Patients #1 and #4 opted for an inclusion of informal caregiver to assist with tDCS administration.

At the initial visit at the patient’s home, patients and co-participating informal caregivers provided informed consent and were screened. Initial familiarization with the device proceeded without difficulties and patients received size-fitted headbands for electrode placement for the following montages: the “M1-SO” with anode on the left (Patient #1) or right (Patient #3); and the “DLPFC” montage (Patients #2 and 4). All patients underwent the initial acceptability test and found the intensity of stimulation at 1.5 mA acceptable. Training proceeded without difficulty and all designated tDCS operators (Patients #2 and 3 themselves and assisting caregivers for Patients #1 and #4), passed the competency test and were allowed to proceed with tDCS applications.

In the first tDCS phase, all patients received 10 applications on 10 consecutive days. In the second “as needed” phase, Patients #1 and 3 did not opt for additional applications during the subsequent period and Patients #2 and 4 received another 10 sessions, no more than one session per day. No adverse events were reported.

Outcome data collection using the CMSAS was carried out without problems. There were no missing data and the results illustrate feasibility of tracking symptom scores. At the baseline, Patients #1 and 2 had high Global Distress Score of 32.4 and 39.2, respectively; the score substantially declined to 9.6 and 14.0, respectively after 10 consecutive tDCS applications, and remained low, 4.0 and 11.0, respectively, at the end of the as needed phase in which Patient #1 did not opt for any tDCS session and Patient #2 received 10 additional applications. Patients #3 and 4 had the baseline Global Distress Score low, 8.6 and 8.0, respectively, and no substantial change was noted in the course of tDCS treatment; the score was 7.6 and 8.6, respectively, after 10 tDCS applications, and 11.6 and 10.0, respectively, after the optional phase in which Patient #3 has not received any tDCS application and Patient #4 opted for 10 sessions. These results do not reflect efficacy given the small sample, lack of a comparator, and varied number of tDCS applications; they do, however, demonstrate the ability of symptom measurement to capture different response profiles during home-based tDCS trials.

**FIGURE 5:**
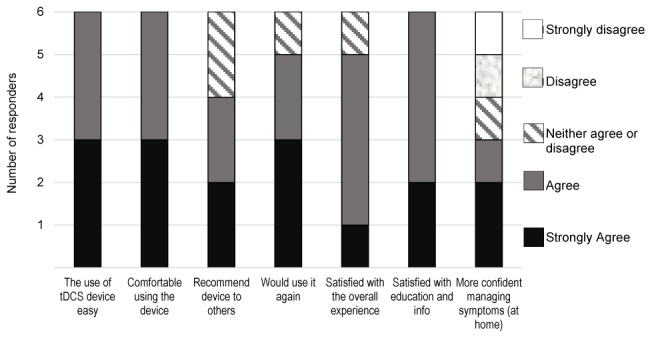
Results of the tDCS satisfaction survey by the four patients and two co-participating caregivers.

On the satisfaction survey (**Figure 5**), all four patients and the two co-participating caregivers indicated “agreed/strongly agreed” in response to statements “I find the use of tDCS device easy,” “I was comfortable using the tDCS device,” and “I was satisfied with the education and information I received before using the device.” Five users agreed/strongly agreed with statements “If I were offered this device and equipment in the future I would use it again,” and “I was satisfied with the overall experience of using the tDCS device,” four users would recommend the device to others, and three users agreed/strongly agreed with statement “With the help of the tDCS device I am more confident managing the symptoms at home.”

## Discussion

We have developed a novel at-home tDCS intervention with telehealth support and have demonstrated initial feasibility in four patients with complex symptom control problems associated with chronic illness. There were no difficulties with the initial study visit at the patient’s home, training or patients’ acceptance of the procedure. The adherence to the protocol was excellent; no session required discontinuation, all patients received 10 tDCS sessions as recommended in the initial phase and proceeded to the as-needed phase. Data collection was without problems and there were no missing data. Although a decrease of post-tDCS CMSAS scores was noted in two patients, no conclusions should be drawn from these findings as there was no control comparison, the number of subjects was low, and the study was not designed to evaluate efficacy of the procedure. Regardless, the patients’ and caregivers’ satisfaction with the procedure was high; they were satisfied with the training and were comfortable using the device, indicating very good acceptability of the intervention. Overall, our findings suggest that with proper training and enhanced remote monitoring, tDCS application in home-bound patients with multiple chronic symptoms is possible.

The approach allows for tailoring the procedures to the needs and preferences of the patient, as well as includes elements for enhanced safety and adherence monitoring, low burden, and ease of use. The instructional materials, training procedures, and quality check enable patients and their informal caregivers to develop skills necessary for tDCS application in accordance with good practices. The frequent contact with a tDCS technician via video-conference and telephone enables remote assistance as needed and enhances safety and adherence monitoring. Safety and adherence with the protocol are further supported by technical features of the equipment, which includes remote dose control, and simple easy-to-use headgear allows for reliable electrode montage without performing the 10–20 EEG measurements.

Importantly, this novel intervention comports with guidelines for tDCS use in clinical patient-populations in home settings (Charvet et al., 2015). The recommendations include: (1) training of staff in tDCS treatment and supervision; (2) assessment of the user’s capability to participate in tDCS remotely; (3) training procedures and materials including assessments of the user and/or caregiver; (4) simple and fail-safe electrode preparation techniques and tDCS headgear; (5) strict dose control for each session; (6) ongoing monitoring of compliance/adherence to the protocol; (7) monitoring for treatment-emergent adverse effects; and (8) guidelines for discontinuation of a session and/or study participation (Charvet et al., 2015).

This novel non-pharmacological adjuvant tDCS-telehealth intervention is well positioned to address the needs of seriously ill patients in home settings and promote the national goal of more effective community-based care. It can also support the efforts to reduce the need for drug therapy, particularly in chronically ill older patients. Recent surveys indicate that almost one-third of Medicare beneficiaries take ≥5 prescribed medications, and the polypharmacy is associated with higher risks for the patients when possible adverse effects of drug therapy may compound the risks associated with disease-related organ dysfunction (Centers for Medicare and Medicaid Services, 2012; Hasselman and Center for Health Care Strategies, 2013; Ortman et al., 2014). Although existing evidence pertaining to the tDCS potential to reduce medication intake stems from studies in postoperative settings (Borckardt et al., 2011, 2013; Glaser et al., 2016), this tDCS-telehealth intervention can facilitate future explorations of this potential for the chronically ill in home settings.

Each component of this novel intervention, tDCS and the telehealth support, has potential to substantially facilitate symptom control. Randomized controlled studies (Boggio et al., 2008; Hagenacker et al., 2014; Fagerlund et al., 2015; Lefaucheur, 2016; and others) have shown that tDCS delivered in several sessions can reduce symptoms common in chronic illness, such as pain, fatigue, sleeping difficulties, cognitive difficulties, worrying, or depression, and can result in decreased demand for symptom-directed medications (Borckardt et al., 2011, 2013; Glaser et al., 2016), while telehealth support may help the chronically ill get engaged in symptom monitoring, facilitate access to information about symptom management, or facilitate contact with the support team (Kearney et al., 2009; Kroenke et al., 2010; Head et al., 2011; Johnston et al., 2012; Ruland et al., 2013; and others). In the past decade, various telephone-based or computer-based systems have been tested to enhance reporting and monitoring symptoms in patients with serious illnesses, such as cancer, in both urban and rural areas, and preliminary results suggest high acceptance of this approach by patients and families (Davis et al., 2007; Weaver et al., 2007; Grubaugh et al., 2008; Kroenke et al., 2010; Head et al., 2011). Although it is impossible to circumvent the physical and psychological impact of chronic illness on patients and their families, telehealth technology provides innovative remote ways toward improving patient education, symptom assessment, communication and outreach by the support team.

Further, the protocol for application of this at-home tDCS-telehealth intervention can serve as a template or a generic protocol for future tDCS studies or tDCS clinical applications in the chronically ill. Various sets of outcome assessment tools can be easily incorporated in the protocol and the number of tDCS sessions in the two phases of tDCS applications can be adjusted as well. The tDCS-telehealth intervention offers many opportunities for further modification of the technology and the protocol to the needs of various patient populations.

Overall, we aimed for the development and initial feasibility testing of the novel tDCS-telehealth intervention for home-bound chronically ill patients with multiple unrelieved symptoms. The pilot study was not intended to investigate the clinical efficacy of the intervention and no conclusions about the efficacy should be drawn from the reported results. Further, due to a low sample size, the findings on feasibility of the procedure in the home-bound chronically ill are preliminary, and larger studies are needed to further evaluate the intervention and to determine/optimize the protocol efficacy and effectiveness for symptom control outcomes.

## Author Contributions

AR contributed to the data collection, data evaluation, design of graphic materials, and the manuscript writing, with important intellectual input from the rest of the team. VP contributed to the development of instructional materials. BP contributed to the technical solutions. RP contributed to the development of design of the protocol and the manuscript writing, with important intellectual input from the rest of the team. MB contributed to the technical solutions and manuscript writing, with important intellectual input from the rest of the team. HK lead the design and development of the overall project, and contributed to the data evaluation and the manuscript writing, with important intellectual input from the rest of the team.

## Conflict of Interest Statement

MB has equity in Soterix Medical Inc. The City University of New York has patents on brain stimulation with MB as co-inventor. The other authors declare that the research was conducted in the absence of any commercial or financial relationships that could be construed as a potential conflict of interest.
